# Gill Adaptation of the Hong Kong Catfish ( *Clarias fuscus* ) to Chronic Heat Stress: Tissue Remodeling, Enhanced Antioxidant Defense and Immune Metabolism Regulation

**DOI:** 10.1002/ece3.73470

**Published:** 2026-04-14

**Authors:** Cunyu Duan, Yong Liu, Fuxin Liu, Dayan Zhou, Yulei Zhang, Guangli Li, Cailin Huang, Chuanhao Pan, Huapu Chen, Changxu Tian

**Affiliations:** ^1^ Fisheries College, Guangdong Ocean University Guangdong Engineering Technology Research Center of Indigenous Valuable Fish Reproductive Regulation and Breeding, Guangdong Provincial Key Laboratory of Aquatic Animal Disease Control and Healthy Culture Zhanjiang China; ^2^ Guangxi Introduction and Breeding Center of Aquaculture Nanning China

**Keywords:** catfish, histological structure, molecular regulation, oxidative resistance, thermotolerance

## Abstract

Rising temperature fluctuations associated with global climate change pose an increasing threat to fish survival. This study investigated the adaptive mechanisms in the gills of Hong Kong catfish ( *Clarias fuscus* ) under long‐term high temperature stress. *C. fuscus* were cultured at natural‐temperature (NT, 26°C) and high‐temperature (HT, 34°C) for 90 days, with histopathological, biochemical, and transcriptomic differences analyzed between the two groups. Histopathological findings revealed that continuous HT treatment reduced the gill surface area and induced apoptosis. Biochemical analyses showed that prolonged HT treatment significantly increased the activities of superoxide dismutase (SOD, *p* = 0.0281), catalase (CAT, *p* = 0.0388), and glutathione peroxidase (GPX, *p* = 0.0394), while reducing malondialdehyde (MDA, *p* = 0.0312) level. The status of the antioxidant system and the survival time may be associated with the re‐establishment of antioxidant balance and an overall enhancement of the fish's antioxidant capacity. Transcriptome analysis indicated that long‐term HT treatment induced significant expression changes in 675 genes, of which 407 were significantly upregulated and 208 were significantly downregulated. These differentially expressed genes (DEGs) were primarily enriched in the biological processes concerned with immune response and energy metabolism. The results suggest that *C. fuscus* enhanced thermal resilience by modulating immune pathways to mitigate excessive apoptosis and upregulating energy metabolism to support tissue repair. The findings demonstrate that although chronic heat stress induces structural damage in the gills of *C. fuscus* , it simultaneously strengthens the fish's antioxidant defenses, improves stress tolerance, and activates repair mechanisms. This study provides a valuable molecular and physiological foundation for understanding the adaptive evolution of fish in a warming environment and provides guidance for the implementation of effective species protection measures.

## Graphical abstract

Continuous high‐temperature culture altered the structure of gill tissue, enhanced antioxidant capacity, and promoted apoptosis *C. fuscus*. In response, the organism mitigates extensive apoptosis and strengthens its repair capacity by regulating immune and energy metabolism pathways. This research lays a foundation for future explorations into the thermal tolerance plasticity of fish and provides insights into effective species diversity protection strategies to cope with the challenge of global warming.

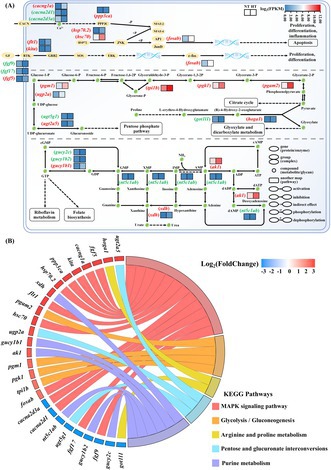

## 1 Introduction

The rapid advancement of scientific and technological capabilities over recent decades has accelerated industrial development, deepening the imbalance between humanity and nature. This imbalance has contributed to ongoing global warming and an increase in the frequency of extreme climate events (Barbarossa et al. 2021). By the end of this century, temperature changes are projected to become more intense, frequent, and persistent (Meehl and Tebaldi 2004). One critical environmental consequence of climate warming is the rise in water temperatures, which threatens aquatic biodiversity and the stability of the aquatic ecosystems (Kuan et al. 2022). As a key component of aquatic life, fish are particularly vulnerable to temperature changes. Within their tolerance range, they can adapt to varying temperatures by regulating their physiological functions (Messina et al. 2023). However, when temperature fluctuations exceed this specific range, physiological dysfunction may occur (Khieokhajonkhet et al. 2022; Liu et al. 2016, 2022). The accumulation of stress responses can ultimately lead to tissue damage or even death, posing an inevitable challenge to the survival of aquatic organisms (Chen et al. 2021). At the same time, research has shown that fish may reshape their stress tolerance by activating adaptive regulatory mechanisms under sustained stress conditions (He et al. 2023). Among these mechanisms, changes in tissue morphology, the regulation of the antioxidant system, and immune and metabolic responses under stress may play a key role in shaping the acquired tolerance of fish (Dalvi et al. 2017; Islam et al. 2022; An et al. 2010). Therefore, studying the histological changes, enzymatic responses, and molecular mechanisms in fish under continuous high‐temperature (HT) culture conditions provides a research foundation for understanding the regulatory processes and adaptive mechanisms of fish under thermal stress.

Gill serves as the primary organ for gaseous interchange between the fish's body and its aquatic environment, playing a crucial role in maintaining ion balance. However, because it is fully exposed to the water, gills are especially susceptible to environmental stressors (Wentworth et al. 2018). To mitigate the adverse effects of these stressors, gills undergo tissue morphological remodeling and exhibit various immune and antioxidant responses at the cellular level (Esam et al. 2022; Wu et al. 2017). In the study of triploid rainbow trout ( *Oncorhynchus mykiss* ), it was observed that the body compensates for chronic heat stress by increasing the exposed surface area of the gills in water, thereby ensuring sufficient oxygen supply for metabolic regulation and anti‐inflammatory effects (Ma et al. 2024). However, excessively HTs or prolonged exposure can lead to gill tissue damage. An experiment on *Lophiosilurus alexandri* revealed that after 35 days of heat stress at 32°C, notable disruptions in the gill lamellae, ruptures of the gill epithelium, and increased cell proliferation occurred (Takata et al. 2018). Similarly, in grass carp ( *Ctenopharyngodon idella* ), it was found that when the temperature exceeded the optimal range, excessive reactive oxygen species (ROS) activated the antioxidant system, upregulating the expression of antioxidant enzymes in gill tissue to combat the stressful environment (Khan et al. 2024). The study of *Cyprinus carpio* indicated that chronic heat stress led to heightened expression of the NF‐κB signaling pathway in gill tissue, enhancing the immune response while maintaining cellular homeostasis through degrading damaged proteins and inhibiting apoptosis (Cheng et al. 2024). Given that fish gill tissue is particularly sensitive to aquatic stressors, it may serve as the most immediate indicator of environmental stress impact (Shen et al. 2024). Investigating the alterations in gill morphological structure, antioxidant responses, and molecular mechanisms following prolonged HT stress can provide clearer views into the adaptive capacity and heat tolerance of the organism under chronic heat stress.

The Hong Kong catfish ( *Clarias fuscus* ) is commonly found in tropical and subtropical freshwater environments south of the Yangtze River in China. As the only member of the Clariidae fish native to China, it demonstrates significant market potential (Tian et al. 2023; Hu and Li 2016). By dint of its conspicuous suitability and resilience to stress, *C. fuscus* has become one of the preferred commercial fish for large‐scale cultivation in South China (Xu et al. 2021; Lin et al. 2022). However, the physiologic status of *C. fuscus* is adversely affected by the substantial increase in water temperature associated with ongoing global warming. Previous experimental studies on temperature treatment have shown that prolonged exposure to HT environment impacts *C. fuscus* liver tissue structure and immune metabolism (Liu et al. 2024), and alters its response mechanisms to acute heat stress (Duan et al. 2024). Given the crucial role of gill tissue in mediating interactions between the organism and its environment, the capacity of *C. fuscus* gills to respond to chronic heat stress also warrants significant research attention.

Current research on HT stress in fish predominantly employs short‐term treatment methods (Kim et al. 2019; Cheng et al. 2018; Wang et al. 2019). However, long‐term thermal stress tests more accurately reflect an organism's response to environmental pressures and are better aligned with the actual conditions of biological evolution. In this study, we investigate the adaptive regulatory mechanisms of gill tissue in *C. fuscus* under prolonged thermal stress. Through histological examinations, enzyme activity assays, and transcriptomic analysis, we aim to offer a comprehensive understanding of heat tolerance plasticity in fish. This research not only enhances our knowledge of adaptive evolution in the context of global warming and provides practical insights into the thermal tolerance plasticity of fish, but also provides guidance for the implementation of effective species protection measures.

## 2 Materials and Methods

### 2.1 Ethics Statement

The experimental protocols used in this study were approved by the Animal Research Ethics Committee of Guangdong Ocean University (NIH Pub. No. 85‐23, revised 1996). No protected or endangered species were included in the study.

### 2.2 Animals

The 1‐month‐old fish with an average weight of 1 ± 0.2 g were sourced from Guangxi Hongtai Aquatic Product Farm, Guangxi Province, China. The experimental temperature and duration were controlled in accordance with previously established methods (Liu et al. 2024). After a week of temporary culture in the freshwater aquaculture base of Guangdong Ocean University, *C. fuscus* were randomly assigned to culture under two temperature conditions for a duration of 90 days: three natural‐temperature (NT) groups (26°C ± 2°C, NT group) and three HT groups (34°C ± 0.5°C, HT group). Each parallel group contained 300 fish. For the HT group, the initial water temperature was set at 26°C and subsequently increased at a rate of 1°C per hour. Once the temperature reached 34°C, it was maintained at that level until the end of the experiment. During the test, the pH of the water was maintained between 6.8 and 7.5, dissolved oxygen concentrations ranged from 6.2 to 7.8 mg/L, and ammonia nitrogen levels were kept within 0.05–0.15 mg/L. These conditions were controlled to eliminate the potential influence of other environmental stressors on the experimental outcomes (Li et al. 2025). Each group was fed twice daily at 9:00 and 17:00. The feeds used were No. 1 and No. 2 floating pellet feeds of Fenghua brown yellow croaker feed (crude protein ≥ 36%, lysine ≥ 1.7%, crude fat ≥ 5.0%, crude fiber ≤ 8.0%, crude ash ≤ 15.0%, total phosphorus ≤ 0.5%). The nutritional components met the requirements of standardized aquaculture to eliminate feed interference (Li et al. 2025). At the conclusion of the culture period, gill tissues of six individuals in two temperature groups were collected respectively. All sampling operations were performed under anesthesia with ethyl m‐aminobenzoate methanesulfonate (MS‐222). Before each fish sampling operation, the tools were disinfected with alcohol and HT burning (Liu et al. 2024; Tawfeek et al. 2024). For subsequent testing, the tissues were promptly preserved in Bouin's solution and stored in liquid nitrogen at −80°C.

### 2.3 Histopathological and Apoptosis Analysis of Tissue

Gill samples fixed within Bouin's solution for 24 h were subsequently dehydrated, embedded and sliced. The histological alterations in the gills were examined using hematoxylin–eosin (HE) staining, while apoptosis was assessed TdT‐mediated dUTP Nick‐End Labeling (TUNEL) staining. Under the fluorescence microscope, DAPI stained the nucleus of all cells blue, while FITC stained the nucleus of apoptotic cells red.

### 2.4 Antioxidant Index Detection

ELISA detection kits (Enzyme‐linked Biotechnology, Shanghai, China) were used to quantify the activities of catalase (CAT), superoxide dismutase (SOD), glutathione peroxidase (GPX) activities, and malondialdehyde (MDA) levels, following the manufacturer's instructions. All samples were subjected to both biological and technical replicates to minimize variability arising from individual differences and procedural inconsistencies.

### 2.5 Molecular Mechanism Exploration

RNA extraction, library construction, sequencing, and assembly were performed in accordance with previously established procedures (Liu et al. 2024). The reference genome index (NCBI Accession: PRJNA1173847) provides access to the RNA‐Seq data. Subread was employed to quantitatively analyze the expressing‐levels across all samples, while DESeq2 was utilized to identify differentially expressed genes (DEGs) with a *p*_adj_ < 0.05 and |log_2_FoldChange| ≥ 2.0 (Liao et al. 2014; Varet et al. 2016). Subsequently, clusterProfiler was applied to investigate the enrichment of DEGs in Gene Ontology (GO) terms and KEGG pathways (Yu et al. 2012).

### 2.6 Quantitative Real‐Time PCR Confirmation

To confirm the authenticity of the transcriptome screening results, we verified 10 genes using qRT‐PCR. The primer sequences designed by Primer Premier 5 are shown in Table 1. Total RNA was extracted and reverse‐transcribed using the previously described experimental procedures (Liu et al. 2024). Subsequent qRT‐PCR cycle index consisted of: 94°C for 180 s, 35 cycles of 94°C for 30 s, 60°C for 30 s, and 72°C for 30 s. To ensure complete primer extension, the reaction was maintained at 72°C for an additional 600 s. Finally, the expression variations of genes between the two groups were ascertained by 2^−ΔΔ*Ct*^ method with *β‐actin* as the standard.

**TABLE 1:** Primers used for qRT‐PCR in *C. fuscus* .

Gene ID	Gene name	Primer sequences(5′‐3′)	Accession number
Cfus03868	*β‐Actin*	F: AGGCTGGATTCGCTGGAGATGAT R: TGGTGACAATACCGTGCTCAATGG	PZ195022
Cfus16255	*col11a1*	F: GGGAGTACGACATGAGCGAG R: GTTGACCGTTAGCACCTGGA	PZ195023
Cfus10242	*sod3*	F: GAACTCGTACCTGACCGCAA R: CCACCCCCAAGGGATTGTAG	PZ195024
Cfus21137	*nfkb2*	F:ACGTTCGGAGGAAACAGTCC R:TGACGAGGAGTAGCCGAAGA	PZ195025
Cfus01346	*ido2*	F: TGCTAAACCAGGCTGACGAG R: TGATGAAGCGTGACACGACA	PZ195026
Cfus22446	*slc25a25b*	F: AAGCGACTGATCGGAAGCAA R: GGGCGAGACGAGTCTTCAAA	PZ195027
Cfus21065	*psip1*	F: TCAGCACCTGCAGAAACACA R: ACTGATCAGCGTCTCTCCCT	PZ195028
Cfus07570	*camk2g*	F:TGTCTGCGCAAGTTCAATGC R: TCTCCTTCAGGGCACTCACT	PZ195029
Cfus04867	*slc25a38*	F: ATCCCCGGTGTGGGAATCTA R: AGTTATAACGCCCGCTCTCG	PZ195030
Cfus17451	*ice1*	F: GGCTGGAAATGGACCCATGA R: TCTCGGACTGGTGTTTGCTC	PZ195031
Cfus19226	*sh3bgr*	F: ACATGCAGATCACCATGGCA R: ACTGTGCGAAATCGTGTCCT	PZ195032

*Note:* Each primer pair was designed based on the reference sequence corresponding to the accession number of the last column.

### 2.7 Statistical Analysis

All statistical analysis in this experiment were performed utilizing the unpaired *t*‐test of GraphPad Prism 9. The data were expressed as mean ± SD (*n* = 6), and *p* < 0.05 was considered statistically significant.

## 3 Results

### 3.1 Effects of Chronic Heat Stress on Gill Tissue Structure and Apoptosis

**FIGURE 1:**
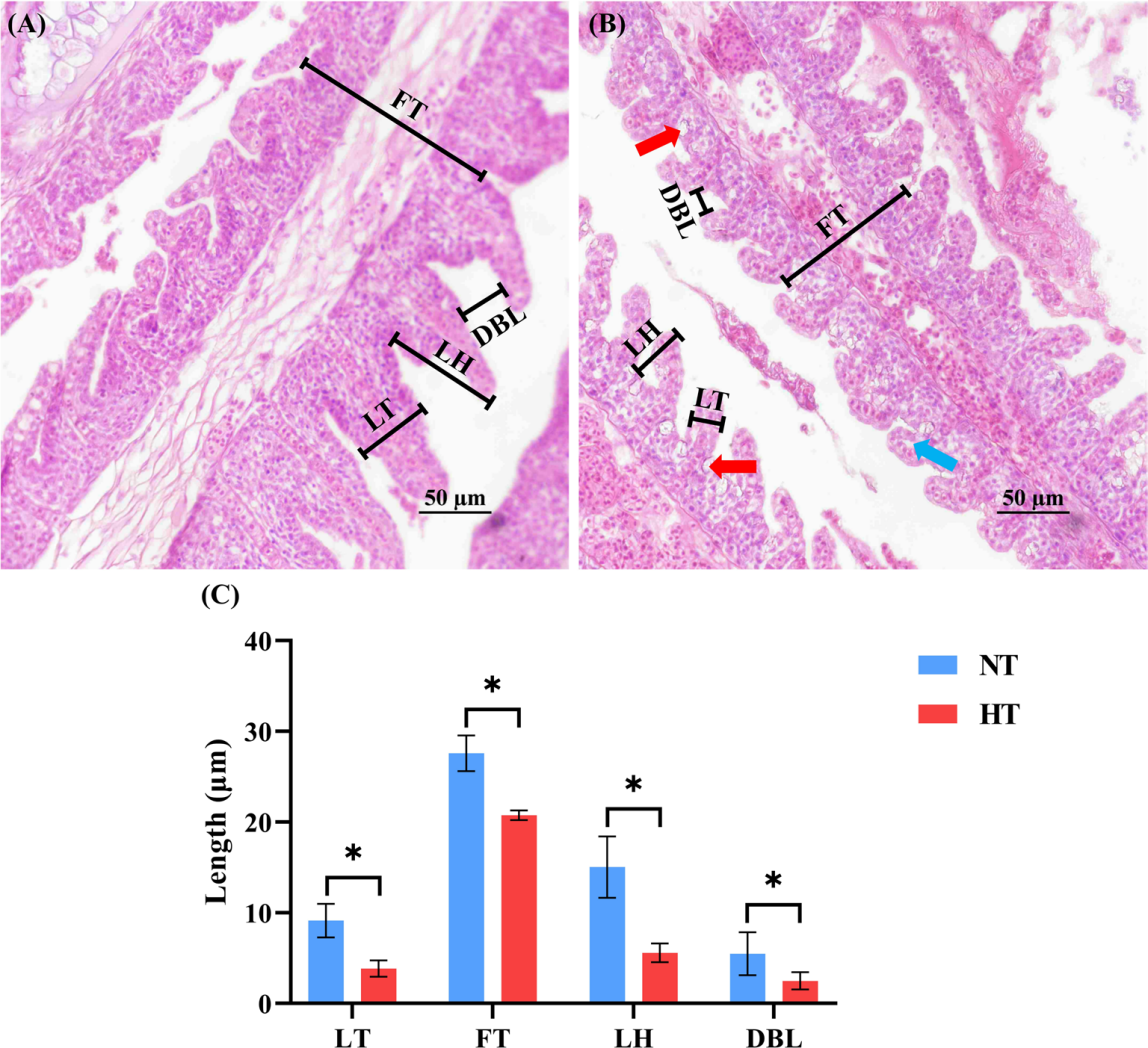
Analysis of gill tissue structure of *C. fuscus* under chronic thermal stress. (A) Gill tissue structure of the NT group; (B) Gill tissue structure of the HT group; (C) Comparison of morphological measurement parameters between the NT and HT groups, expressed as mean ± SD (*n* = 6). An asterisk (*) indicates a significant difference between the NT and HT groups (*p* < 0.05). DBL, Distance between lamellas; FT, Filament thickness; LH, Lamellar height; LT, Lamellar thickness.

**FIGURE 2:**
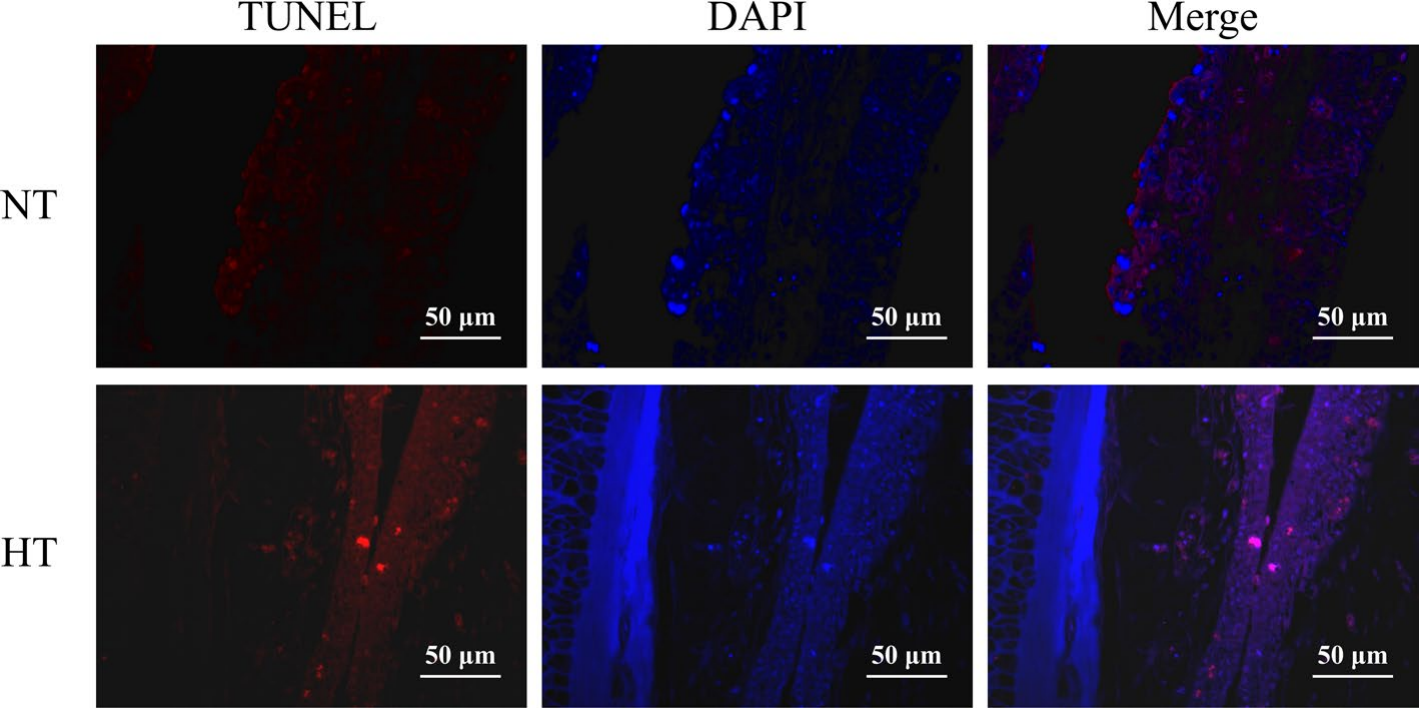
The apoptosis of gill tissue in NT and HT groups.

Through HE and TUNEL staining experiments, it was showed that continuous HT exposure significantly affected the gill structure and physiological state of *C. fuscus* . Following prolonged exposure to heat stress, epithelial elevation and mucus hypertrophy were observed in the gill lamellae. Concurrently, a reduction in the gill filament thickness (FT), lamellae thickness (LT), lamellae height (LH), and the distance between lamellae (DBL) was observed (Figure 1). In addition, TUNEL staining showed red fluorescence signals in apoptotic cells, while DAPI staining showed blue fluorescence signals in all cells, including both apoptotic and non‐apoptotic cells. Combined with the two staining results, it was found that the number of apoptotic cells in the gill tissue of the HT group increased relative to the NT group (Figure 2).

### 3.2 Effects of Chronic Thermal Stress on Antioxidant Indices of Gill Tissue

**FIGURE 3:**
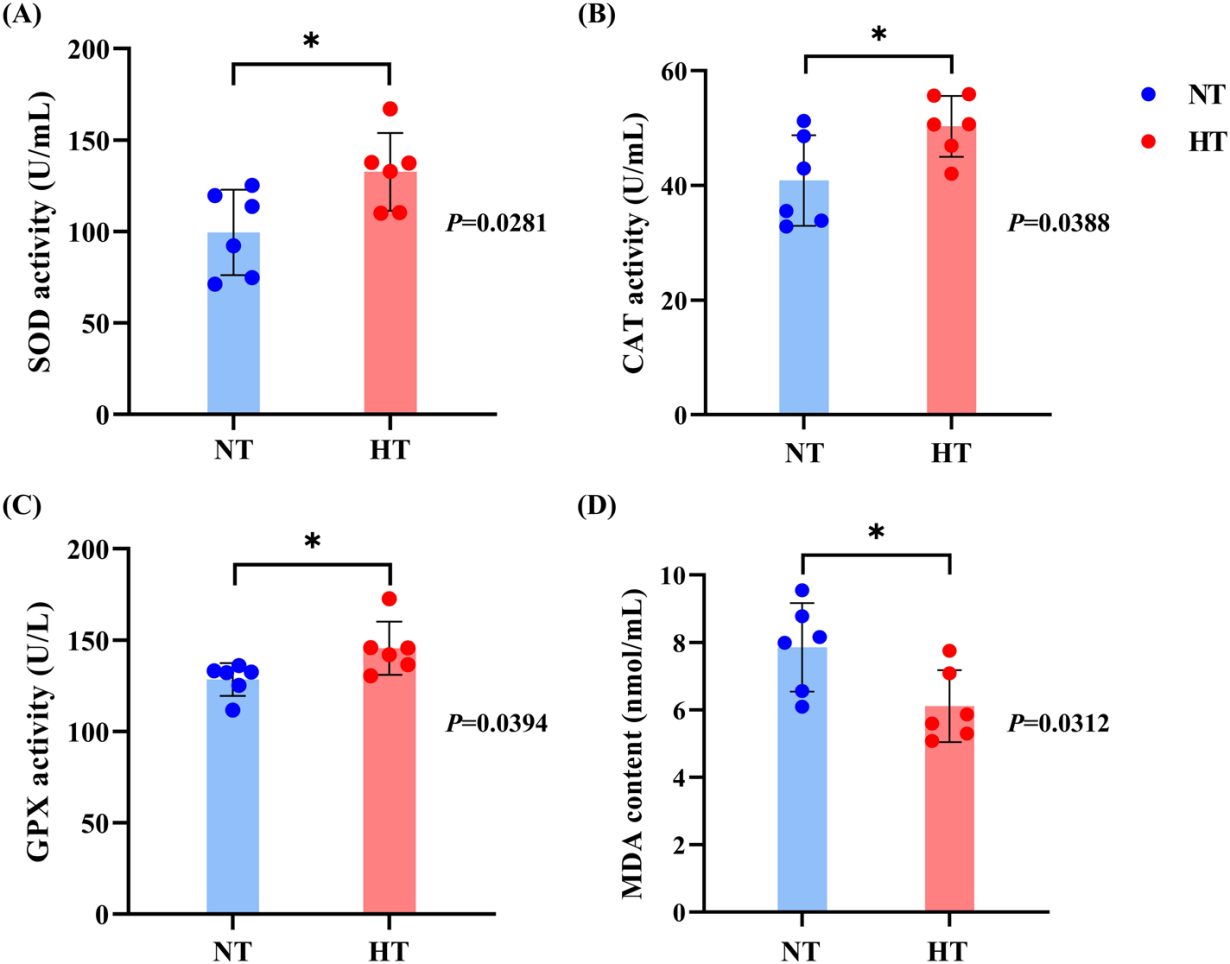
Comparison of antioxidant parameters in gill tissue between NT and HT groups. (A) Comparison of SOD activity between NT and HT groups. (B) Comparison of CAT activity between NT and HT groups. (C) Comparison of GPX activity between NT and HT groups. (D) Comparison of MDA content between NT and HT groups. Values are expressed as mean ± SD (*n* = 6). An asterisk (*) indicates a significant difference between the HT and NT groups (*p* < 0.05).

The levels of antioxidant indices in gill tissues of the NT and HT groups are presented in Figure 3. Compared to the NT group, the activities of SOD, CAT, and GPX in the HT group were significantly increased (*p* < 0.05, Figure 3A–C). Correspondingly, the MDA content in the HT group was significantly decreased (*p* < 0.05, Figure 3D). Among them, the activity of SOD showed the most significant difference between the two groups (*p* = 0.0281, Figure 3A), followed by MDA content (*p* = 0.0312, Figure 3D).

### 3.3 Analysis of Transcriptome Sequencing

#### 3.3.1 Analysis of DEGs

**FIGURE 4:**
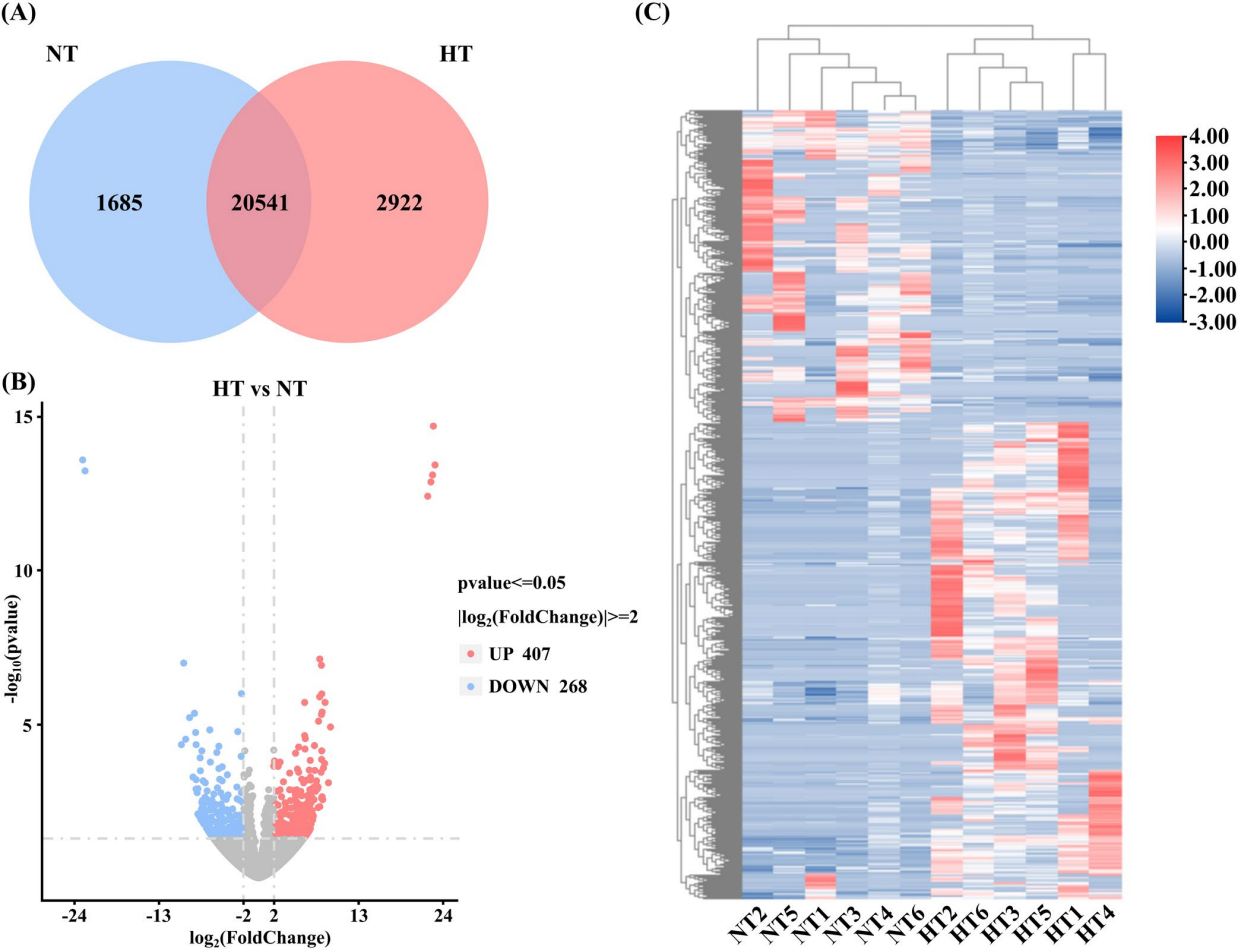
(A) The overlap of expressed genes between the NT and HT groups. (B) Number of DEGs, with upregulation and downregulation in the HT group compared to the NT group. Red indicates upregulation and blue indicates downregulation. (C) Hierarchical clustering analysis of DEGs between the NT and HT groups. Gene expression levels range from high (red) to low (blue).

The results displayed that an amount of 25,148 genes were expressed in both the NT and HT group, with 20,541 genes coexpressed across these groups. Specifically, 1685 genes were uniquely expressed in the NT group, while 2922 genes were uniquely expressed in the HT group (Figure 4A). A total of 675 DEGs were identified, comprising 407 upregulated genes and 268 downregulated genes (Figure 4B). Notably, the number of upregulated genes in the HT group surpassed that of the downregulated genes. The heat map revealed that, although there are some intragroup differences among the parallel samples, a significant overall clustering indicates substantial differences in the expression patterns between the two gene groups (Figure 4C).

#### 3.3.2 DEGs Enrichment Analysis

**FIGURE 5:**
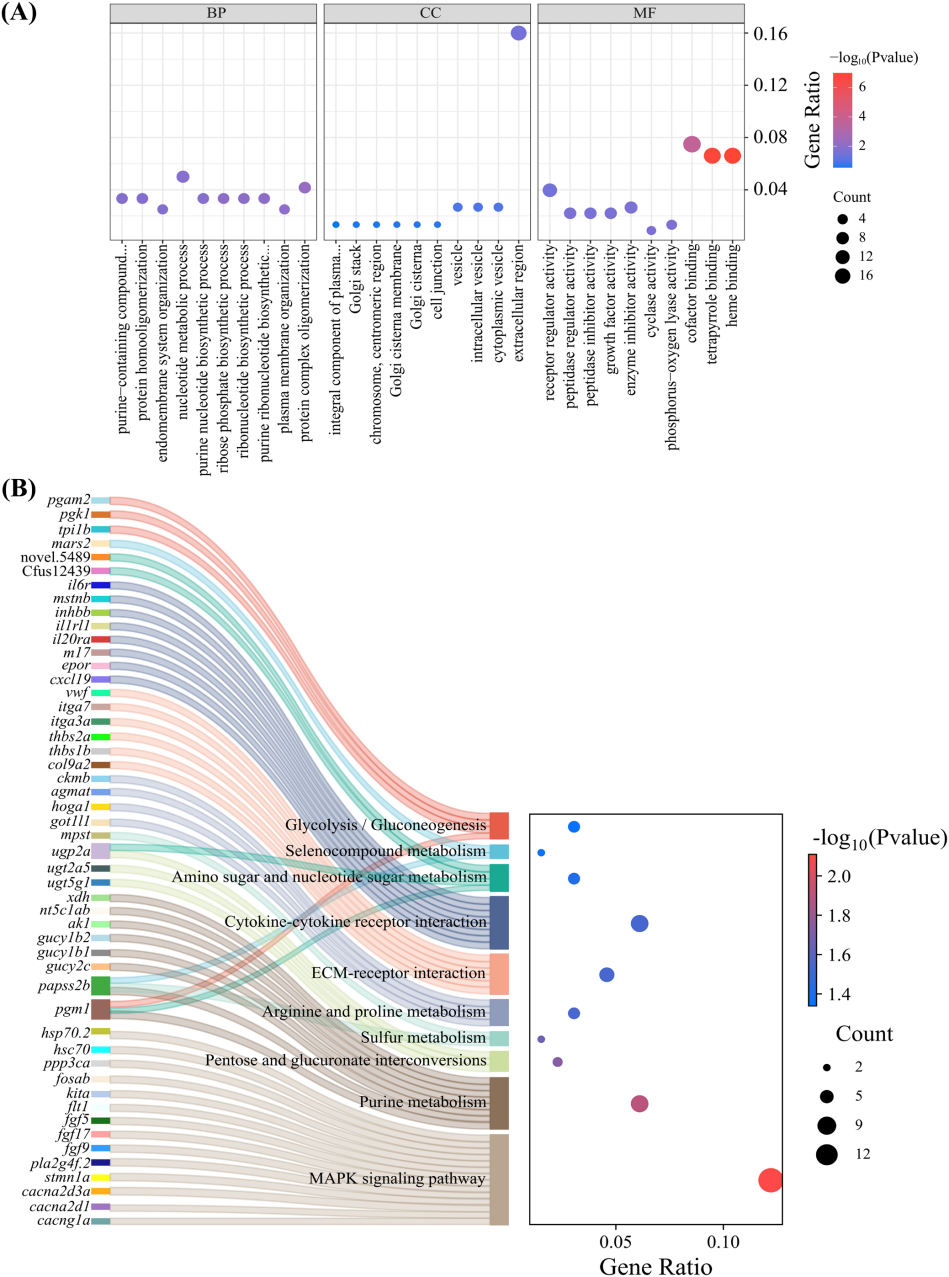
(A) GO functional enrichment analysis of DEGs, displaying the top ten enriched terms in Biological Process (BP), Cellular Component (CC), and Molecular Function (MF) categories. (B) KEGG pathway enrichment analysis of DEGs, highlighting the top ten enriched pathways and the associated genes. The significance of enrichment is color‐coded from high (red) to low (blue).

A total of 519 GO terms were enriched from 675 DEGs, comprising 284 biological process terms (BP), 57 cellular component terms (CC) and 178 molecular function terms (MF). Further analysis revealed that the DEGs were significantly enriched in GO terms such as heme binding, tetrapyrrole binding, cofactor binding, and protein complex oligomerization (Figure 5A). KEGG analysis indicated that 110 pathways were enriched. The top 10 pathways included the MAPK signaling pathway, purine metabolism, pentose and glucuronate interconversions, sulfur metabolism, arginine and proline metabolism, ECM‐receptor interaction, cytokine‐cytokine receptor interaction, amino sugar and nucleotide sugar metabolism, selenocompound metabolism, and glycolysis/gluconeogenesis (Figure 5B).

#### 3.3.3 Apoptosis, Antistress and Energy Metabolism Pathway Regulation Analysis

**FIGURE 6:**
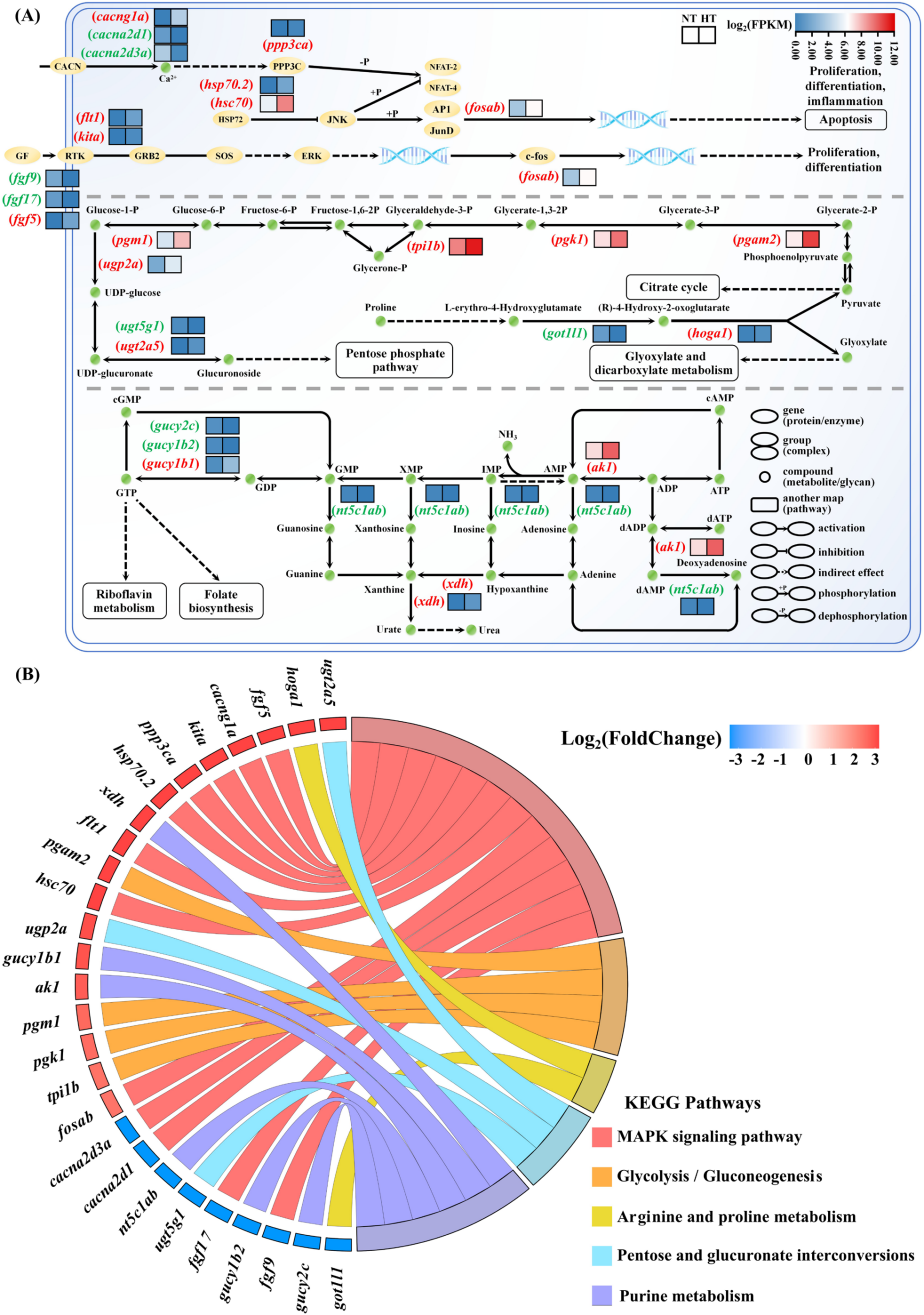
(A) The MAPK signaling pathway, glycolysis/gluconeogenesis, pentose and glucuronate interconversions, arginine and proline metabolism and purine metabolism (part). Upregulated genes are shown in red, and downregulated genes are shown in green. The log_2_(FPKM) values range from blue (low) to red (high). (B) Comparison of key DEGs in the aforementioned five pathways between the NT and HT groups. The log_2_(FoldChange) values are ranked from high to low, displayed from red (high) to blue (low).

Enrichment analysis revealed significant differences in immune metabolism between the NT and HT groups. To further explore the specific regulatory mechanisms in the gill tissue of *C. fuscus* cultured at different temperatures, we analyzed the hub DEGs' expression differences in major immune and metabolic processes. The results showed that, compared to the NT group, the HT group exhibited significantly increased expression of *ppp3ca* (related to immune promotion), as well as *hsp70.2* and *hsc70* (associated with apoptosis inhibition). Additionally, genes linked to cell proliferation and differentiation, such as *fgf5*, *flt1*, *kita*, and *fosab*, were upregulated. Conversely, *fgf9* and *fgf17* were downregulated. In the glycolysis and gluconeogenesis pathways, the expression levels of *pgm1*, *tpi1b*, *pgk1*, and *pgam2* were significantly upregulated. Similarly, in the glycolytic pathway linked to the pentose phosphate pathway, *ugp2a* and *ugt2a5* were also significantly upregulated. During proline metabolism, *got1l1* was downregulated while *hoga1* was upregulated. In purine metabolism, *nt5c1ab* (involved in the formation of xanthine and hypoxanthine) was downregulated, whereas *xdh* (involved in the conversion of xanthine and hypoxanthine to urea) was significantly upregulated. Additionally, *gucy1b1* (catalyzing the conversion of GTP to cGMP) was upregulated, while *gucy2c* and *gucy1b2* were significantly downregulated, potentially leading to increased GTP content and indirectly promote the synthesis of riboflavin that can alleviate oxidative stress (Figure 6).

### 3.4 qRT‐PCR Validation

**FIGURE 7:**
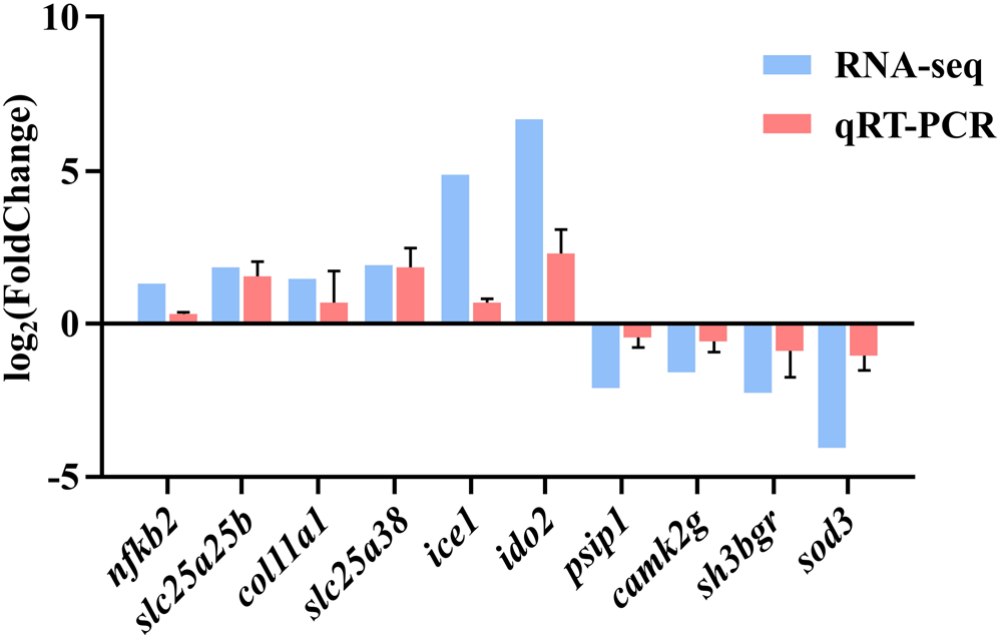
Comparative analysis of qRT‐PCR results and transcriptome data.

Ten genes were randomly selected for validation by qRT‐PCR. The expression patterns of all selected genes were consistent between qRT‐PCR and RNA‐seq results (Figure 7), elucidating the precision and dependability of RNA‐seq data.

## 4 Discussion

In recent years, global warming has intensified discussions regarding how teleost fish adapt to rising water temperatures. Fluctuations in water temperature can disturb the cellular processes and homeostasis of fish (Jeong et al. 2021). Previous studies have confirmed that chronic thermal stress can induce oxidative damage to the liver and trigger protective mechanisms in the body (Liu et al. 2024). However, the capacity of the gills to respond to chronic heat stress remains unclear. This study conducted long‐term HT treatments to observe histological changes, apoptosis, enzyme activity, and transcriptomic alterations in the gills of *C. fuscus* , with the aim of elucidating the response mechanisms of gill tissue to chronic thermal stress and assessing the adaptability of *C. fuscus* to such conditions.

### 4.1 Long‐Term HT Treatment Alters Gill Morphology and Promotes Apoptosis

The gill tissue, as an organ directly exposed to the external environment, is more sensitive to ambient temperature fluctuations than other organs (Khieokhajonkhet et al. 2022; Shin et al. 2018). Histopathology serves as a crucial indicator for directly reflecting the degree of environmental impact on fish (Ma et al. 2020). Former researches have revealed that heat stress provokes gill damage in various species, including coho salmon ( *Oncorhynchus kisutch* ) (Clark et al. 2012), *Paralichthys olivaceus* and its hybrids ( *P. olivaceus* ♀ × *P. dentatus* ♂) (Liu et al. 2017), as well as the redbelly yellowtail fusilier *Caesio cuning* and five‐lined cardinalfish *Cheilodipterus quinquelineatus* (Johansen et al. 2021). In this study, prolonged exposure to HTs resulted in significant tissue damage in the gill tissue of *C. fuscus* , characterized by epithelial uplift of lamellae, mucus hypertrophy, and reductions in FT, as well as LT, LH, and spacing. These changes may reduce the contact area between the gill tissue and the aquatic environment, serving as a protective mechanism to prevent further disruption of the internal homeostasis caused by environmental stressors. However, this reduction in contact area may compromise the oxygen uptake necessary for the organism's metabolic activity (Chen et al. 2021). Further analysis revealed that continuous HT treatment led to an increase in apoptotic cells, which could impair the physiological functions of the tissue (Xv et al. 2024).

### 4.2 Long‐Term HT Treatment Modulates Oxidation Balance and Enhances Antioxidant Capacity

HT stress disturbs the oxidative and antioxidant balance in fish, leading to increased free radical production. Excessive free radicals can induce oxidative damage, triggering a cascade of further free radical production and subsequent damage (Rahal et al. 2014). Although a normal level of ROS is crucial for maintaining cell functions, stress‐induced imbalances between ROS production and clearance result in elevated MDA levels, indicating tissue damage (Chen et al. 2022; Cheng et al. 2018; Dalvi et al. 2017). Antioxidant enzymes such as SOD, CAT, and GPX play important roles in ROS scavenging. These enzymes typically act in concert to protect the body from oxidative stress (Li et al. 2019). In this study, we observed that continuous HT stress significantly increased the activities of SOD, CAT, and GPX in the gill tissues of the HT group compared with the NT group, while MDA levels significantly decreased. This suggests that heat stress activates the antioxidant system, reduces MDA accumulation, and establishes a new oxidative equilibrium under prolonged HT conditions, thereby mitigating oxidative damage (Dong et al. 2022). These findings are correspond with research outcomes on *Onychostoma macrolepis* , *Genypterus chilensis* , and *Labeo rohita* , which also demonstrated that heat stress can enhance antioxidant defenses (Yu et al. 2017; Dettleff et al. 2020; Roychowdhury et al. 2021).

### 4.3 Long‐Term HT Treatment Regulates Immune and Energy Metabolism Pathways

Transcriptome sequencing technology is extensively used to investigate environmental impacts at the biomolecular level (Zhao et al. 2021). In this study, numerous genes were specifically expressed in either the NT or HT group, with significant variations in the expression of DEGs between the two groups. This suggests that continuous thermal treatment may significantly alter the molecular mechanisms that maintain normal physiological functions in the gill tissue of *C. fuscus* , thereby mitigating the negative effects of heat stress (Paul and Small 2021). GO and KEGG analyses revealed significant differences between HT and NT groups in immune and energy metabolism related pathways. These findings align with research on Russian sturgeon ( *Acipenser gueldenstaedtii* ), indicating that organisms can restore internal homeostasis under stress by regulating the immune metabolic pathways (Costábile et al. 2022).

Apoptosis, as one form of programmed cell death, is crucial for maintaining internal stability by eliminating damaged or infected cells, and plays a key role in cell development and immune processes (Luo et al. 2017; Cheng et al. 2015). The MAPK signaling pathway, a central mediator of apoptosis, also plays a significant role in cellular responses to HT stress (Zhu et al. 2024). Within this pathway, Ppp3ca, a catalytic subunit of calcineurin A, dephosphorylates nuclear factors of activated T cells (Nfat), allowing them to enter the nucleus and interact with activating protein‐1 (Ap‐1) to enhance immune responses (Im and Rao 2004; Karagiota et al. 2019; Xu et al. 2022). Hsp72, a protective protein with low basal expression but high expression under stress, mitigates the negative effects of stress by inhibiting JNK activity (Levada et al. 2018). In another signaling cascade, growth factors (Gf) bind to and activate receptor tyrosine kinases (Rtks), ultimately regulating cell fate (Katz et al. 2007). In this study, the expression levels of *ppp3ca*, *hsp70.2*, *hsc70*, and *fosab* were significantly increased. This suggests that after long‐term thermal treatment, *C. fuscus* enhances its immune response and prevents large‐scale apoptosis. The significant changes in the expression of *fgf5*, *fgf9*, *fgf17*, *flt1*, and *kita* indicate that the molecular mechanism regulating cell fate have adapted to the elevated temperatures. These findings are correspond with researches on scallop ( *Argopecten irradians* ), where heat stress leads to significant changes in the phosphorylation of JNK and ERK within the MAPK pathway, potentially balancing apoptosis and cell production under heat stress conditions (Li, Chang, et al. 2024).

Purine is a vital component in organisms, crucial for numerous cellular processes (Chen et al. 2023). Studies have indicated that purine metabolism has a regulatory effect on immune response under stress conditions, protecting the organism from damage (Li, Li, et al. 2024). In this study, the downregulation of *gucy2c* and *gucy1b2*, along with the upregulation of *gucy1b1*, suggests an accumulation of guanosine triphosphate (GTP) within the purine metabolic pathway. This accumulation may enhance riboflavin metabolism and folate biosynthesis, thereby boosting energy metabolism, immune function, and antioxidant capacity in *C. fuscus* under prolonged thermal stress (Wang et al. 2025; Xing et al. 2022). In addition, the upregulation of *xdh* in transcriptional xanthine oxidase promotes the conversion of hypoxanthine to xanthine and xanthine to urate (Baldissera et al. 2017). This finding is similar to the changes in xanthine oxidase activity observed during oxidative damage in the gills of largemouth bass ( *Micropterus salmoides* ) induced by sodium carbonate peroxyhydrate (Sinha et al. 2020). Conversely, the downregulation of *nt5c1ab* reduces the conversion of adenosine monophosphate (AMP) and guanine monophosphate (GMP) to hypoxanthine and xanthine (Maiuolo et al. 2016). Additionally, the upregulation of *ak1* increases the conversion rate of AMP to adenosine triphosphate (ATP) (Kulkarni et al. 2011), indicating an adaptive response to mitigate and repair oxidative damage.

Energy metabolism is crucial for maintaining normal physiological activities under stress, encompassing carbohydrate, lipid, and amino acid metabolism (Shan et al. 2019; Huo et al. 2019). In this study, DEGs were significantly enriched in glycolysis/gluconeogenesis, pentose and glucuronate interconversions, and arginine and proline metabolism pathways after continuous HT stress. During glycolysis/gluconeogenesis, the balance between glucose utilization and storage is key to maintaining blood glucose levels and ensuring a sustained energy supply (Yang et al. 2024). Pentose and glucuronate interconversions also play a critical role in energy metabolism and are involved in the biotransformation processes, aiding in the degradation and excretion of toxic metabolites (Zhou et al. 2023; Wang et al. 2020). The significant upregulation of *pgm1*, *tpi1b*, *pgk1*, and *pgam2* suggests an increased activity of glucose metabolism‐related enzymes to meet the energy demands under HT (Wang et al. 2024). The upregulation of *ugp2a* and *ugt2a5*, together with the downregulation of *ugt5g1*, indicates an enhanced detoxification process, likely in response to the increased production of harmful substances under heat stress, thereby balancing energy metabolism and mitigating adverse effects. Proline, involved in maintaining mitochondrial respiration, cellular protection, and improving disease resistance and heat adaptability, shows altered metabolism under stress (Vosloo et al. 2013; Zhao et al. 2015). The downregulation of *got1l1* in arginine and proline metabolism pathway may slow proline decomposition, while the upregulation of *hoga1* promotes the production of pyruvate and glyoxylate, both of which enter the TCA cycle to generate energy (Wu et al. 2011).

Collectively, these results show that *C. fuscus* adapts to HT by regulating pathways related to immune and energy metabolism. On the one hand, it alleviates oxidative stress damage caused by heat stress; on the other hand, it provides sufficient energy for body repair. However, this experiment primarily examined the effect of 90‐day fixed HT treatment on the gill tissue of *C. fuscus* , without fully accounting for the gradual and fluctuating nature of water temperature rise under climate change. In the natural environment, HT rises slowly and exhibits diurnal fluctuations. Future studies should simulate the effects of frequent temperature fluctuations by incorporating temperature gradient designs. In addition, the temperature tolerance of fish is affected by its developmental stage. In this study, the materials used to explore the temperature tolerance of *C. fuscus* were approximately 1‐month old. The metabolic rate, antioxidant capacity, and heat stress response mechanisms may differ between juvenile and adult fish. Therefore, the research results carry certain limitations. In the future, it is necessary to study the temperature tolerance across different developmental stages to better understand the adaptive potential of *C. fuscus* in response to climate change.

## 5 Conclusion

Overall, continuous HT culture altered the structure of gill tissue, enhanced antioxidant capacity, and promoted apoptosis in *C. fuscus* . In response, the organism mitigates extensive apoptosis and strengthens its repair capacity by regulating immune and energy metabolism pathways. Our study provides valuable information for exploring the histopathological characteristics, biochemical indicators, and specific molecular mechanisms that enable *C. fuscus* to adapt to temperature variations. This research lays a foundation for future explorations into the thermal tolerance plasticity of fish and offers perspectives on effective strategies for species diversity protection to cope with the challenge of global warming.

## Author Contributions

**Cunyu Duan:** conceptualization (equal), data curation (equal), formal analysis (equal), investigation (equal), methodology (equal), validation (equal), visualization (equal), writing – original draft (equal). **Yong Liu:** conceptualization (equal), data curation (equal), formal analysis (equal), investigation (equal), methodology (equal), validation (equal), visualization (equal). **Fuxin Liu:** data curation (equal), investigation (equal). **Dayan Zhou:** data curation (equal), funding acquisition (equal). **Yulei Zhang:** conceptualization (equal), investigation (equal). **Guangli Li:** conceptualization (equal), investigation (equal). **Cailin Huang:** data curation (equal), investigation (equal). **Chuanhao Pan:** data curation (equal), investigation (equal). **Huapu Chen:** conceptualization (equal), data curation (equal), investigation (equal), supervision (equal). **Changxu Tian:** conceptualization (equal), data curation (equal), funding acquisition (equal), investigation (equal), methodology (equal), supervision (equal), writing – review and editing (equal).

## Funding

This work was supported by Guangdong Basic and Applied Basic Research Foundation, 2025A1515012346, 2026A1515011409. Department of Education of Guangdong Province, 2023KTSCX042. Zhanjiang Science and Technology Plan Project, 2025R02111. Undergraduate Innovation Team Project of Guangdong Ocean University, CXTD2023003. Youth Science and Technology Innovation Talent of Guangdong TeZhi plan talent, 2023TQ07A888. Guangdong Provincial Special Fund for Modern Agriculture Industry Technology Innovation Teams, 2024CXTD26.

## Ethics Statement

The experimental protocols used in this study were approved by the Animal Research Ethics Committee of Guangdong Ocean University (NIH Pub. No. 85‐23, revised 1996). No protected or endangered species were included in the study.

## Conflicts of Interest

The authors declare no conflicts of interest.

## Data Availability

The datasets generated in this study have been uploaded to the NCBI database with the accession number PRJNA1173847.
